# Short-term cortisol exposure alters cardiac hypertrophic and non-hypertrophic signalling in a time-dependent manner in rainbow trout

**DOI:** 10.1242/bio.037853

**Published:** 2018-10-19

**Authors:** Karoline S. Nørstrud, Marco A. Vindas, Göran E. Nilsson, Ida B. Johansen

**Affiliations:** 1 Department of Biosciences, University of Oslo, Oslo 0371, Norway; 2 Department of Food Safety and Infection Biology, Faculty of Veterinary Medicine, Norwegian University of Life Sciences, Oslo 0454, Norway

**Keywords:** Relative ventricular mass, Cell proliferation, Cortisol receptors, Natriuretic peptides, Hypertrophy markers, Acute stress

## Abstract

Cardiac disease is a growing concern in farmed animals, and stress has been implicated as a factor for myocardial dysfunction and mortality in commercial fish rearing. We recently showed that the stress hormone cortisol induces pathological cardiac remodelling in rainbow trout. Wild and farmed salmonids are exposed to fluctuations and sometimes prolonged episodes of increased cortisol levels. Thus, studying the timeframe of cortisol-induced cardiac remodelling is necessary to understand its role in the pathogenesis of cardiovascular disease in salmonids. We here establish that 3 weeks of cortisol exposure is sufficient to increase relative ventricular mass (RVM) by 20% in rainbow trout. Moreover, increased RVMs are associated with altered expression of hypertrophic and non-hypertrophic remodelling markers. Further, we characterised the time course of cortisol-induced cardiac remodelling by feeding rainbow trout cortisol-containing feed for 2, 7 and 21 days. We show that the effect of cortisol on expression of hypertrophic and non-hypertrophic remodelling markers is time-dependent and in some cases acute. Our data indicate that short-term stressors and life cycle transitions associated with elevated cortisol levels can potentially influence hypertrophic and non-hypertrophic remodelling of the trout heart.

## Abstract (precis)

**Summary:** Here we indicate that short-term cortisol exposure can induce cardiac hypertrophic and non-hypertrophic remodelling and that certain pro-hypertrophic effects of cortisol are time-dependent in rainbow trout.

## INTRODUCTION

The salmonid heart is a highly plastic organ, well known for its ability to remodel and grow in response to physiological stimulation (Gamperl and Farrell, 2004). For example, heart growth caused by cold acclimation (Farrell et al., 1988; Vornanen et al., 2005) has been described in several salmonid species and is characterised by cardiomyocyte growth (Vornanen et al., 2005), increased expression of angiogenesis and hypertrophy markers and normal or enhanced contractile function (Graham and Farrell, 1989). The mammalian heart is also capable of extensive remodelling and growth, both in response to physiological (i.e. exercise and pregnancy) and pathological (i.e. pressure overload, inflammatory disease) stimuli (Weeks and McMullen, 2011; Shimizu and Minamino, 2016). Of note, there are marked differences between physiological and pathological heart growth in mammals. Physiological hypertrophy is associated with expansion of the capillary network, normal architecture and organisation of cardiac structure and normal or enhanced pumping capacity. Meanwhile, pathological hypertrophy is associated with capillary rarefaction, fibrotic remodelling and reduced systolic and diastolic function. Distinct signalling pathways mediate the different forms of hypertrophic remodelling in mammals (Wilkins et al., 2004). Therefore, certain signalling molecules can serve as markers of pathological pro-hypertrophic signalling.

Importantly, cardiac disease and deformities are increasing problems in farmed animals, where sudden mortality related to cardiac illness have been demonstrated for example in broiler chicken (Maxwell and Robertson, 1998), cattle (Bradley et al., 1981) and salmonid fishes (Poppe et al., 2007). Causes leading to the development of cardiac disease and abnormalities in production animals are poorly understood, although acute stress has been suggested to trigger cardiac events and mass mortality (Maxwell and Robertson, 1998; Poppe et al., 2007).

We recently demonstrated that 45 days of treatment with the steroid stress hormone cortisol, a well-known pro-hypertrophic stimulant in mammals, resulted in a 34% increase in relative ventricular mass (RVM) in rainbow trout (*Oncorhynchus mykiss*). Interestingly, the ventricular growth coincided with impaired cardiovascular and physical performance and an upregulation of specific molecular markers associated with cardiac hypertrophy and pathology (Johansen et al., 2017). More precisely, cortisol exposure induced increased expression of the cardiac hypertrophy markers slow myosin light chain 2 (*smlc2*) and the heart failure marker atrial natriuretic peptide (*anp*). The cortisol treatment also induced increased expression of regulator of calcineurin 1 (*rcan1*), a marker of pathological pro-hypertrophic nuclear factor of activated T-cells (NFAT)-signalling (Wilkins et al., 2004). Thus, cortisol is a potent pro-hypertrophic stimulus also in salmonid fishes and signalling pathways mediating pathological cardiac hypertrophy appears to be conserved from fish to mammals.

Both wild and farmed salmonids are exposed to fluctuations and, sometimes, prolonged episodes of increased cortisol levels in relation to certain life cycle transitions (Schmidt and Idler, 1962; Barton et al., 1985; Barron, 1986; Carruth et al., 2000). For example, during spawning migration, cortisol levels can be elevated for prolonged periods and concentrations may increase from a basal level of approximately 10 ng ml^−1^ to concentrations as high as 640 ng ml^−1^ (Carruth et al., 2000). In addition, natural and artificial chronic stressors (Barton and Peter, 1982; Maule et al., 1988) can result in cortisol concentrations up to 400 ng ml^−1^ (Strange et al., 1978). Concomitantly, salmonid fishes experience extensive cardiac remodelling in association with spawning migration (Franklin and Davie, 1992; Clark and Rodnick, 1998) and under intensive and potentially stressful rearing conditions (Poppe et al., 2002, 2003). Thus, studying the dynamics and timeframe of cortisol-induced cardiac remodelling in fish is necessary to understand its potential role in such phenomena. Is cortisol a stimulus that directly induces pro-hypertrophic signalling and is this signalling immediately followed/associated with increased ventricular mass? If so, then increased expression of markers of such signalling should be evident early in the time course of exposure and brief episodes of stress could result in remodelling of the heart. Alternatively, effects of cortisol on pro-hypertrophic signalling and heart growth may require long-term stress/cortisol exposure.

Here we first established that 21 days of cortisol treatment is sufficient to increase relative ventricular mass and alter expression of several remodelling markers in rainbow trout. Subsequently, we characterised the time course of changes by subjecting rainbow trout to 2, 7 and 21 days of cortisol treatment. We show that cortisol alters the expression of hypertrophic and non-hypertrophic remodelling markers in a time-dependent manner. Cardiac hypertrophy plays a pivotal role in pathological cardiac remodelling and is an independent risk factor for cardiac morbidity and mortality (e.g. congestive heart failure, acute myocardial infarction and sudden death) in humans (Frohlich, 1990; Messerli and Ketelhut, 1991; Lloyd-Jones et al., 2002). Moreover, mechanisms mediating cardiac hypertrophy appear to be conserved along the vertebrate lineage. Thus, our findings are not only relevant from an animal husbandry point of view, but can also provide biomedical relevance in the pursuit to understand vertebrate heart function/failure mechanisms (Shih et al., 2015).

## RESULTS

### Three weeks of cortisol exposure increases RVM and induces pro-hypertrophic signalling and remodelling in rainbow trout

**Fig. 1.**
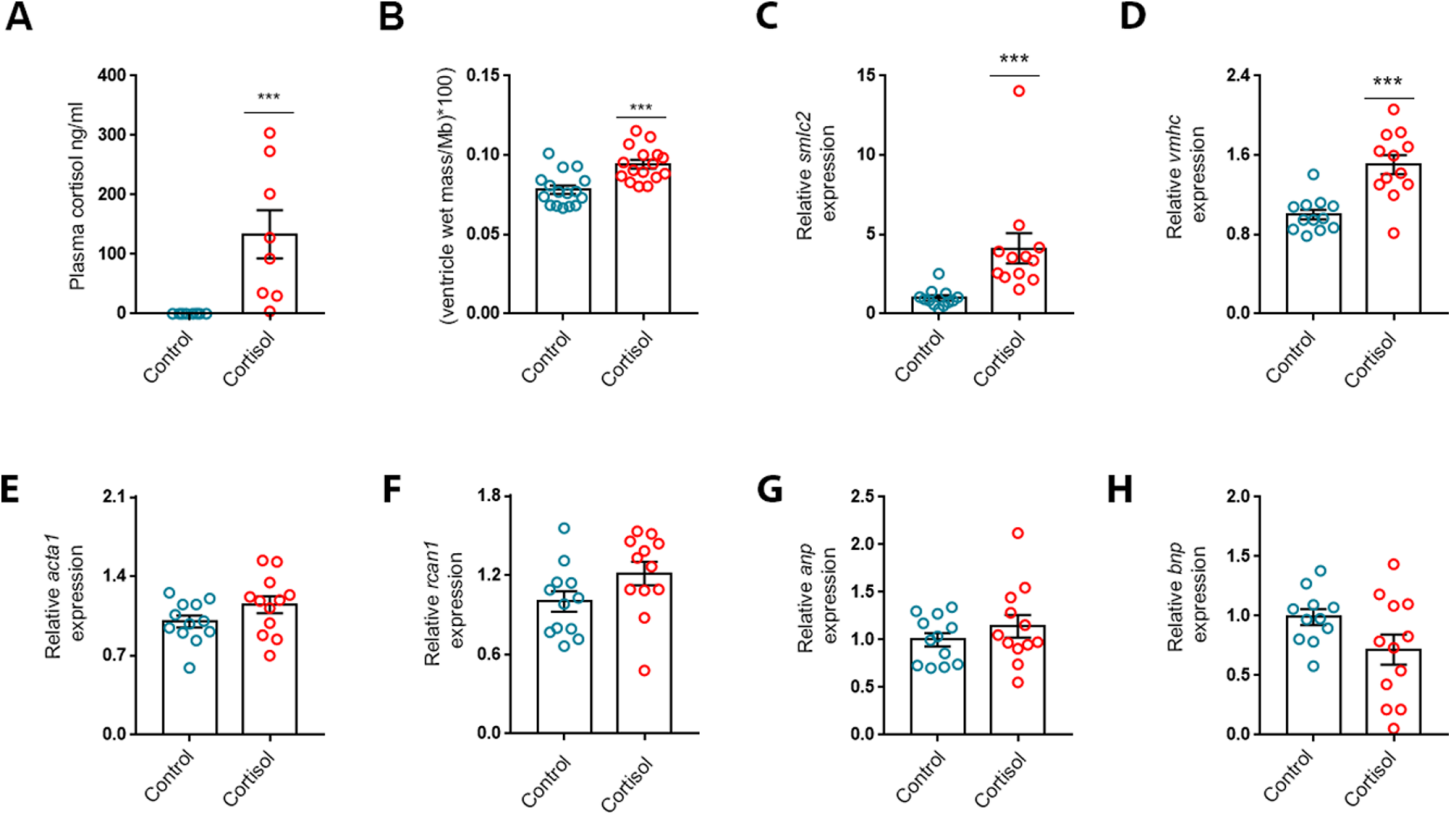
**Three weeks of cortisol exposure increases relative ventricular mass and myocardial pro-hypertrophic signalling in rainbow trout.** (A) Plasma cortisol (*n*=8/group). (B) Relative ventricular mass [RVM; ventricle wet mass/body mass (Mb), *n*=16/group]. (C-H) mRNA abundance of (C) slow myosin light chain 2 (*smlc*2), (D) ventricular myosin heavy chain (*vmhc*), (E) alpha-skeletal actin 1 (*acta1*), (F) regulator of calcineurin 1 (*rcan1*), (G) atrial natriuretic peptide (*anp*) and (H) B-type natriuretic peptide (*bnp*) relative to the standard gene *β-actin* in ventricles of fish treated with cortisol for 21 days (*n*=12/group). Data are either means±s.e.m. (A,B) or means±s.e.m. relative to treatment control (C-H). Mean mRNA expression of treatment control was normalised to 1. Statistical differences were tested by unpaired two-tailed *t*-tests. ***P<0.001 versus control.

To test whether three weeks of cortisol exposure is sufficient to induce cardiac remodelling in rainbow trout, fish were fed cortisol-containing feed for 21 days. Increased plasma cortisol levels were confirmed in cortisol treated fish (Fig. 1A, *n*=8/group). Mean (±s.e.m.) plasma cortisol concentration following 21 days of cortisol treatment resembled concentrations obtained from individuals subjected to chronic stress (Barton, 2002) and was 133.7±40.57 ng ml^−1^, while control fish had a mean (±s.e.m.) value of 0.25±0.05 ng ml^−1^.

In line with previous studies, cortisol treatment resulted in a 20% increase in RVM compared with controls (Fig. 1B, *n*=16/group). Mean (±s.e.m.) RVM was 0.078±0.003 for controls and 0.094±0.003 for cortisol treated fish. The increase in RVMs could, at least partly, be explained by a reduction in body weights. Body weights were reduced in cortisol-treated (219.1±11.5 g) compared to control fish (276.8±10.61 g) after cortisol treatment (unpaired *t*-test; t=3.69, *P*<0.001, data not shown). Absolute ventricle weights were not significantly increased (data not shown).

Still, markers for myocardial pro-hypertrophic signalling were upregulated by the cortisol treatment (*n*=12/group). More specific, *smlc2* (Fig. 1C) and *vmhc* (Fig. 1D) were 4.1- and 1.5-fold increased by the cortisol treatment, respectively. Meanwhile, *acta1* (Fig. 1E), *rcan1* (Fig. 1F) as well as the mammalian heart failure markers, *anp* (Fig. 1G) and *bnp* (Fig. 1H), were not significantly upregulated.

**Fig. 2.**
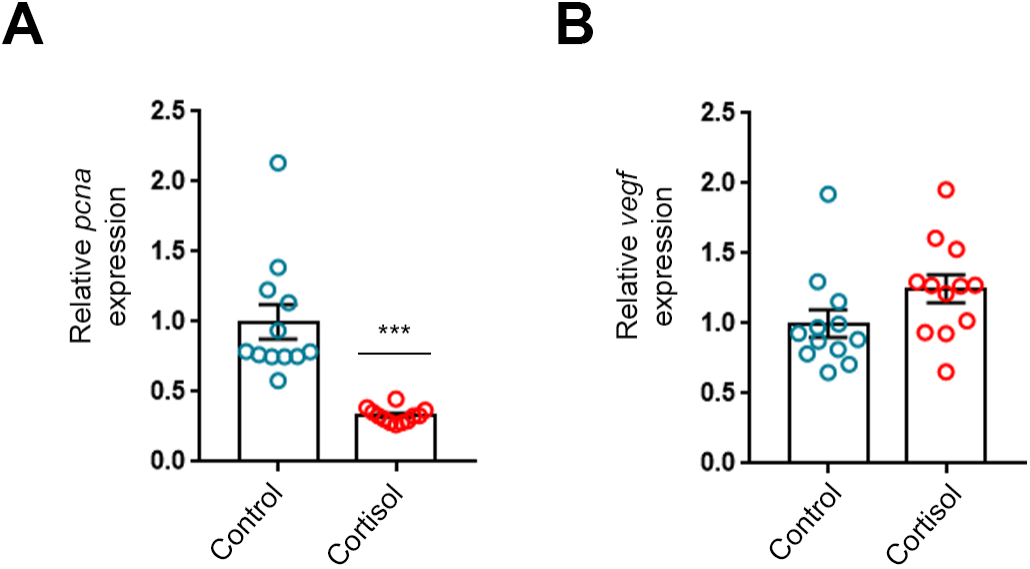
**Three weeks of cortisol exposure reduces myocardial markers of cell proliferation.** The mRNA abundance of (A) proliferating cell nuclear antigen (*pcna*) and (B) vascular endothelial growth factor (*vegf*) relative to the standard gene *β-actin* in ventricles of fish treated with cortisol for 21 days (*n*=12/group). Data are means±s.e.m. relative to treatment control. Mean mRNA expression of control fish were normalised to 1. Statistical differences were tested by unpaired two-tailed *t*-tests. ***P<0.001 versus control.

To indicate non-hypertrophic remodelling of the heart in response to cortisol treatment, the markers of cell proliferation (*pcna*) and angiogenesis (*vegf*) were investigated in the ventricles (Fig. 2, *n*=12/group). The *pcna* mRNA levels were markedly reduced by the cortisol treatment, indicating that cortisol inhibits cell proliferation in the heart at this stage (Fig. 2A). The angiogenesis marker, *vegf*, was not significantly affected by the cortisol treatment (Fig. 2B).

**Fig. 3.**
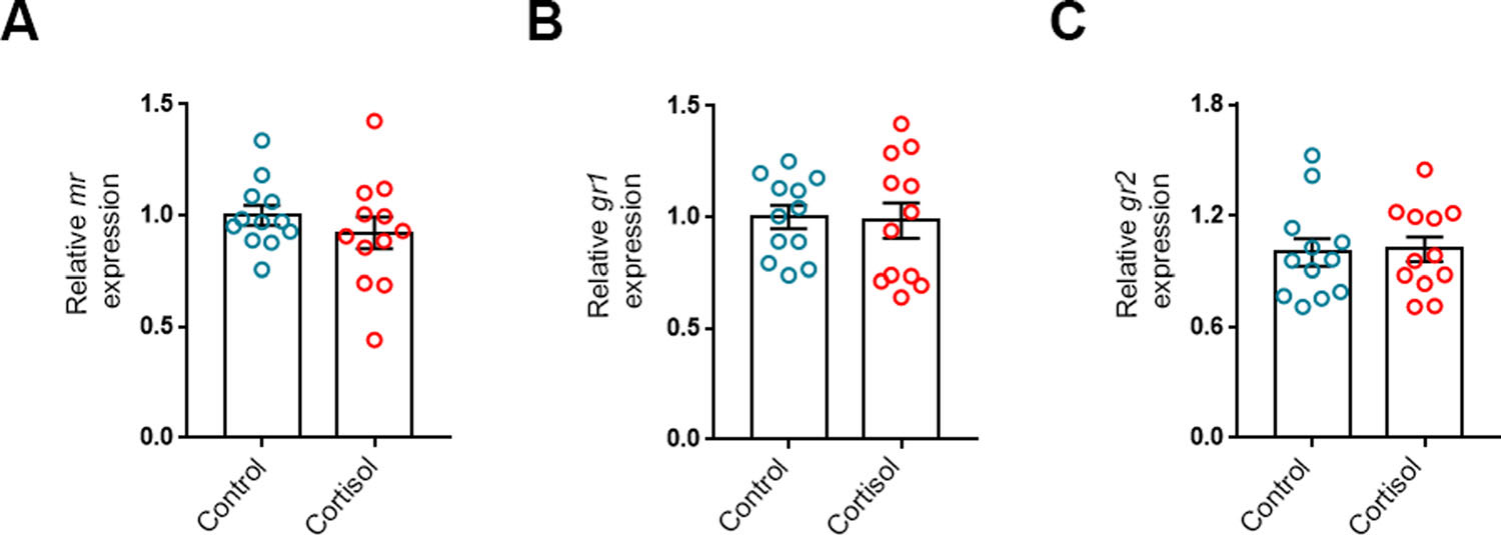
**Three weeks of cortisol exposure does not alter expression of myocardial cortisol receptors.** The mRNA abundance of (A) mineralocorticoid receptor (*mr*), (B) glucocorticoid receptor 1 (*gr1*) and (C) glucocorticoid receptor 2 (*gr2*) relative to the standard gene *β-actin* in ventricles of fish treated with cortisol for 21 days (*n*=12/group). Data are means±s.e.m. relative to treatment control. Mean mRNA expression of control fish were normalised to 1. Statistical differences were tested by unpaired two-tailed *t*-tests.

Three cortisol receptors possibly mediating the actions of cortisol on the heart, are the *mr*, *gr1* and *gr2*. The mRNA expression of these receptors was not significantly altered by 21 days of cortisol treatment (Fig. 3).

### Markers of pro-hypertrophic signalling and remodelling are regulated by cortisol treatment in a time-dependent manner

To investigate the time course of cortisol-induced cardiac remodelling, rainbow trout were treated with cortisol for 2 (*n*_control_=7, *n*_cortisol_=8), 7 (*n*_control_=7, *n*_cortisol_=8) and 21 (*n*_control_=8, *n*_cortisol_=7) days. Data were analysed by two-way ANOVA to assess effects of cortisol treatment, time and whether an average treatment effect was the same for each time point (i.e. interaction effect). Two-way ANOVA was then followed by a Sidak's planned comparison test (with adjusted alpha) to be able to assess differences between treatment groups within each time point.

**Fig. 4.**
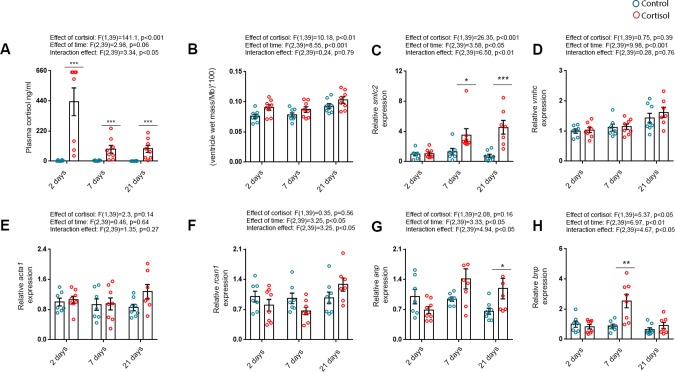
**Markers of pro-hypertrophic signalling and remodelling are upregulated by cortisol treatment in a time-dependent manner.** (A) Plasma cortisol. (B) Relative ventricular mass [RVM; ventricle wet mass/body mass (Mb)]. (C-H) mRNA abundance of (C) slow myosin light chain 2 (*smlc2*), (D) ventricular myosin heavy chain (*vmhc*), (E) alpha-skeletal actin 1 (*acta1*), (F) regulator of calcineurin 1 (*rcan1*), (G) atrial natriuretic peptide (*anp*) and (H) B-type natriuretic peptide (*bnp*) relative to the standard gene *β-actin* in ventricles of fish treated with cortisol for 2 (*n*_control_=7, *n*_cortisol_=8), 7 (*n*_control_=7, *n*_cortisol_=8) and 21 (*n*_control_=8, *n*_cortisol_=7) days. Data are either means±s.e.m. (A,B) or means±s.e.m. relative to 2 days treatment control (C-H). Mean mRNA expression of 2 day control fish were normalised to 1. Statistical differences were tested by two-way ANOVA followed by Sidak's multiple comparison test. *P<0.05 versus control, **P<0.01 versus control, ***P<0.001 versus control.

Overall, cortisol levels were affected by cortisol treatment, but the effect of time (i.e. 2, 7 or 21 days treatment period, *P*=0.06) did not reach statistical significance. However, there was a significant interaction effect between cortisol treatment and time, indicating that cortisol treatment affects plasma cortisol levels in a time-dependent manner. A multiple-comparisons post-hoc test revealed that plasma cortisol levels were higher in cortisol-treated versus respective treatment controls at all time points investigated (Fig. 4A). Mean (±s.e.m.) plasma cortisol concentrations were 2.46±1.32 ng ml^−1^ versus 433.3±101.5 ng ml^−1^ after 2 days, 4.03±1.28 ng ml^−1^ versus 86.79±28.18 ng ml^−1^ after 7 days and 0.80±0.44 ng ml^−1^ versus 98.81±27.74 ng ml^−1^ after 21 days of treatment in control and cortisol-treated fish, respectively.

The observed interaction effect of cortisol treatment and time on plasma cortisol levels was likely related to the fact that feed intake (and hence cortisol administration) was reduced in cortisol-treated fish during the course of the cortisol treatment. Mean±s.e.m daily feed intake was 78.2±6.37, 50.44±7.68 and 37.21±5.36% of daily ration following 2, 7 and 21 days of cortisol treatment, respectively. Mean±s.e.m. daily feed intake in control fish did not decrease and was 61.5±10.54, 83.99±7.53 and 96.13±2.41% of daily ration following 2, 7 and 21 days, respectively (data not shown).

There was a significant effect of both cortisol treatment and time on RVMs of the fish. There was, however, no significant interaction effect between cortisol treatment and time, indicating either that the effect of cortisol on RVM is not dependent on time or alternatively, that time per se affects RVM (Fig. 4B). A multiple comparisons post-hoc test showed that RVMs were not higher in cortisol treated fish versus treatment controls at any of the time points investigated. When processing the data, we noticed that the 21 day treatment controls (mean RVM±=0.093±0.010) had high RVMs compared to the 2 (mean RVM±=0.076±0.015) and 7 (mean RVM=0.078±0.009) day treatment controls and the 21 day treatment controls of group-reared fish (mean RVM=0.078±0.003). To assess whether time (period spent in isolation) affected RVM, we performed a separate post-hoc test comparing RVMs of all treatment controls. Indeed, RVMs of the 21 day treatment controls were significantly higher compared to the 2 day treatment controls (*P*<0.01). Thus, in addition to the effect of cortisol, there was a general trend of RVM increasing over time, which could be incidental or caused by a stress reaction due to being kept in isolation (see Discussion).

There was no significant effect of time [*F*_(2,39)_=2.04, *P*=0.14] or treatment [*F*_(1,39)_=3.96, *P*=0.05] on body weights of the fish, but a significant interaction effect [*F*_(2,39)_=3.55, *P*=0.04]. A post-hoc test revealed that body weights were significantly reduced following 21, but not 2 and 7 days of cortisol treatment (*P*=0.01, data not shown). There was no significant interaction effect [*F*_(2,39)_=2.52, *P*=0.09] or effect of treatment [*F*_(1,39)_=0.08, *P*=0.77] on absolute ventricle weights, but a significant effect of time [*F*_(2,39)_=8.46, *P*<0.001]. Ventricle weights were not different between treatment groups and respective treatment controls at any of the time points investigated (data not shown)

To examine the time course of cortisol effects on pro-hypertrophic signalling, expression levels of *smlc2*, *vhmc*, *acta1*, *rcan1*, *anp* and *bnp* were investigated in the ventricles. There were significant treatment, time and interaction effects on *smlc2* mRNA levels*.* The post-hoc test revealed that *smlc2* mRNA levels were increased following 7 and 21, but not 2 days of treatment, respectively (Fig. 4C). No significant effects were found for the expression levels of *vmhc* and *acta1* mRNA (Fig. 4D-E). The *rcan1* mRNA levels were not significantly affected by cortisol treatment, but there was a significant effect of time and also an interaction effect between the two variables. Post-hoc testing did not, however, reveal significant differences between cortisol treated and treatment controls at any of the time points (Fig. 4F).

There was, however, a time and interaction effect on mRNA levels of both the natriuretic peptides *anp* and *bnp,* but no significant treatment effects. Post-hoc testing revealed a significant increase in *anp* mRNA levels following 21 days of treatment (Fig. 4G) and an increase in *bnp* levels following 7, but not 2 and 21 days of treatment (Fig. 4H).

**Fig. 5.**
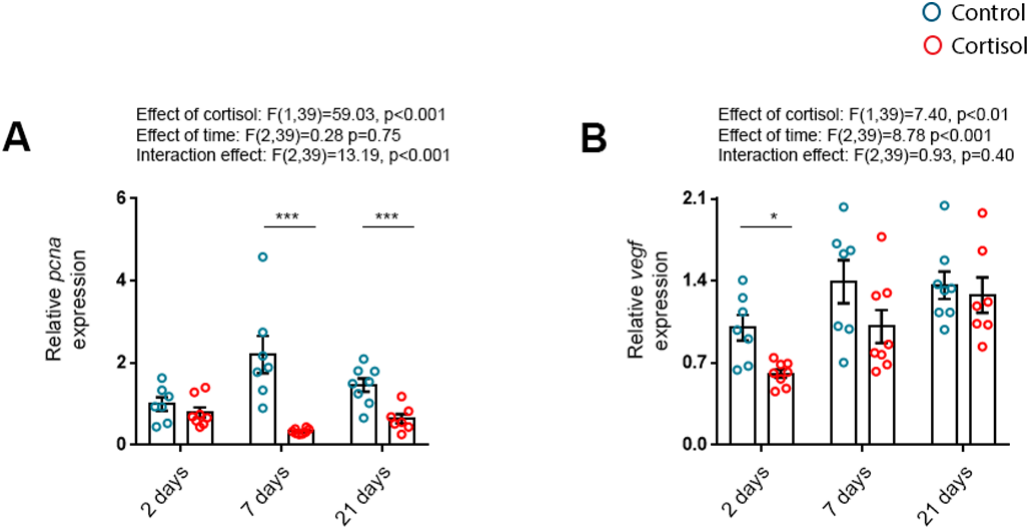
**Markers of non-hypertrophic remodelling are downregulated by cortisol treatment in a time-dependent manner.** The mRNA abundance of (A) proliferating cell nuclear antigen (*pcna*) and (B) vascular endothelial growth factor (*vegf*) relative to the standard gene *β-actin* in ventricles of fish treated with cortisol for 2 (*n*_control_=7, *n*_cortisol_=8), 7 (*n*_control_=7, *n*_cortisol_=8) and 21 (*n*_control_=8, *n*_cortisol_=7) days. Data are means±s.e.m. relative to 2 days treatment control. Mean mRNA expression of 2 days treatment controls were normalised to 1. Statistical differences were tested by two-way ANOVA followed by Sidak′s multiple comparison test. *P<0.05 versus control, ***P<0.001 versus control.

To examine indicators of non-hypertrophic remodelling in the early time-course of cortisol treatment, markers of cell proliferation (*pcna*) and angiogenesis (*vegf*) were investigated in the ventricles (Fig. 5). There was a significant treatment and interaction effect on *pcna* mRNA expression. Specifically, *pcna* was downregulated following 7 and 21 days of treatment (Fig. 5A), supporting that cortisol suppresses myocardial cell proliferation. Expression of *vegf* was also generally reduced by the cortisol treatment and time at the mRNA level, but there was no significant interaction effect. Still, a multiple comparison test revealed significantly reduced *vegf* mRNA levels following 2, but not 7 or 21, days of cortisol treatment (Fig. 5B).

**Fig. 6.**
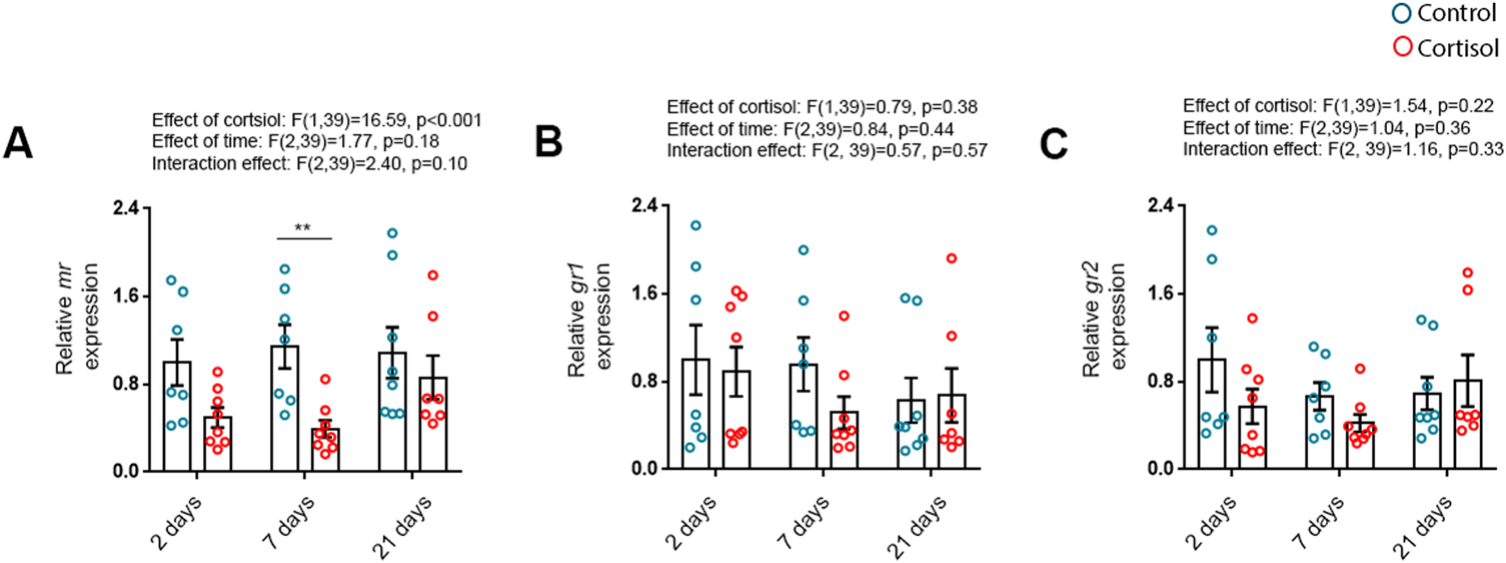
**Cortisol downregulates the expression of myocardial mineralocorticoid receptors.** The mRNA abundance of (A) mineralocorticoid receptor (*mr*), (B) glucocorticoid receptor 1 (GR1) and (C) glucocorticoid receptor 2 (GR2) relative to the standard gene β-actin in ventricles of fish treated with cortisol for 2 (*n*_control_=7, *n*_cortisol_=8), 7 (*n*_control_=7, *n*_cortisol_=8) and 21 (*n*_control_=8, *n*_cortisol_=7) days. Data are means±s.e.m. relative to 2 day treatment control. Mean mRNA expression of 2 day treatment controls were normalised to 1. Statistical differences were tested by two-way ANOVA followed by Sidak's multiple comparison test. **P<0.01 versus control.

Further, to assess the time course of possible effects of cortisol on the expression of its own receptors in the heart, we measured *mr*, *gr1* and *gr2* mRNA levels in the ventricles. Overall, expression levels of *mr* were decreased by the cortisol treatment, but not significantly affected by time. There was no significant interaction effect between the two variables, but a multiple comparison test revealed that *mr* mRNA levels were decreased following 7, but not 2 and 21, days of treatment (Fig. 6A), likely reflecting autoregulation in response to the ligand. No significant effects were found on expression levels of *gr1* and *gr2* (Fig. 6B-C).

## DISCUSSION

In the present work, we show that short-term cortisol exposure alters the expression of a number of cardiac remodelling markers in a time-dependent manner and in support of previous work (Johansen et al., 2017), we show that cortisol treatment increases RVM. Moreover, markers of pro-hypertrophic signalling (i.e. *smlc2*, *vmhc*, *anp* and *bnp*) were upregulated by the cortisol treatment in a time-dependent manner. Both the proliferation marker *pcna* and the angiogenesis marker *vegf* were downregulated during the course of cortisol treatment, indicating that cortisol suppresses myocardial cell proliferation and angiogenesis at an early stage of cortisol exposure. Further, there was a clear tendency for autoregulation of the cortisol receptor *mr* in the cardiac tissue early in the course of exposure, perhaps serving to reduce tissue responsiveness to excess cortisol. Since the observed downregulation of *mr* was not maintained throughout the treatment period, we speculate that such a potentially protective mechanism is temporary and that failure to protect the heart against excess cortisol could partly explain pathological effects of long-term cortisol exposure.

Cortisol exposure for up to 21 days was not sufficient to induce an increase in RVMs comparable to the 34% increase observed previously following cortisol exposure for 45 days (Johansen et al., 2017). Of note and in line with previous findings (Johansen et al., 2017), the cortisol treatment halted somatic growth and a reduction in body weights could, at least partly, explain the observed increase in RVM. Unlike our previous study, where 45 days of cortisol treatment increased absolute ventricle weights, we did not see a significant increase in absolute ventricle weights with treatment for up to 21 days. Thus, pronounced cardiac growth likely require longer exposure time.

Nevertheless, a clear pro-hypertrophic effect of cortisol was indicated by a marked and time-dependent increase in hypertrophy markers. *Smlc2* and *vmhc* were upregulated following 21 days of cortisol treatment of group-reared fish and there were time-dependent increases in *smlc2* (7 and 21 days), *anp* (21 days) and *bnp* (7 days) mRNA expression in the time course study. These data indicate that pro-hypertrophic signalling proceeds hypertrophic growth and an increase in ventricle weight. Of note, we observed a marked cortisol-induced reduction in the expression of cell proliferation marker *pcna* early in the course of cortisol exposure. Reduced cardiac cell proliferation could counteract the pro-hypertrophic effect of cortisol at these stages.

Although increased expression of *vmhc* was only seen in group-reared fish and increased expression of *anp* only in the time course study, increased expression levels of *smlc2*, *vmhc* and *anp* are consistent with previous findings of increased expression in high cortisol responding (HR) fish (Johansen et al., 2011a) and in rainbow trout treated with cortisol for 45 days (Johansen et al., 2017). Increased expression of *bnp*, however, which is a sensitive and commonly used diagnostic marker of elevated cardiac workload and heart failure in humans, has not previously been reported in cortisol-induced cardiac remodelling in rainbow trout. However, *bnp* has been reported to be upregulated during cold-induced adaptive cardiac hypertrophy in rainbow trout (Vornanen et al., 2005; Keen et al., 2015). For example, Vornanen et al. (2005) observed a threefold increase in ventricular *bnp* following 4 weeks of cold-water acclimation. Generally, little data is available concerning regulation, secretion and function of natriuretic peptides associated with cardiac growth and remodelling in non-mammalian species (Tota et al., 2010). However, Vornanen and colleges, argued that since *BNP* serves to protect the mammalian heart by antagonizing the proliferation of cardiac fibroblasts it could serve an adaptive role during cold-induce hypertrophy by protecting the trout heart against the potentially deleterious effects of elevated workload. In our time course study, *bnp* was only transiently increased suggesting that short-term cortisol treatment increases cardiac workload and stresses the salmonid heart to express *bnp*, but that the signal is reduced with prolonged cortisol exposure. Since we know that chronic cortisol exposure eventually induces myocardial hypertrophy, focal fibrosis (Johansen et al., 2011a) and impairs cardiac function (Johansen et al., 2017) in rainbow trout, it is tempting to speculate that a failure to persistently produce and secrete BNP with prolonged cortisol exposure makes the heart vulnerable to the harmful effects of cortisol on cardiac remodelling and function. Similar expression patterns of natriuretic peptides are seen in mammalian models of acute ventricular overload (Hama et al., 1995; Lear and Boer, 1995), hypertension (Wolf et al., 2001) and volume overload (Lear and Boer, 1995). Like in our study, these conditions lead to rapid and sometimes transient increases in *bnp* mRNA expression in the ventricle. Upregulation of *bnp* mRNA is then followed by *anp* upregulation at later time points, similar to what we see in our study. These natriuretic peptides are not normally expressed in the healthy adult mammalian heart. Instead, *anp* and *bnp* are part of the foetal gene program. Re-initiation of the foetal gene program is a common feature of various pathological conditions where the heart experiences extensive remodelling. Other hallmarks of this re-initiation are the switches in isoform expression of genes for sarcomeric proteins such as β-myosin heavy chain (MHC/Myh7, mammalian homologue of *vmhc*), *smlc2* and *Acta1*. It was recently confirmed that a similar isoform switch occurs in response to pathogenic stimulation in the zebrafish (*Danio rerio*) heart (Shih et al., 2015). From our current findings of increased *vmhc* and *smlc2* combined with increased expression of the natriuretic peptides, it is possible that cortisol exposure directly or indirectly triggers an induction of the foetal gene program in rainbow trout.

We previously found that cortisol exposure, in addition to upregulating members of the foetal gene program (e.g. *anp*, *vmhc* and *smlc2*), induces pro-hypertrophic NFAT signalling in the rainbow trout heart (Johansen et al., 2017). The *rcan1* gene is a direct target of the transcription factor NFAT and therefore a mammalian marker of pathological pro-hypertrophic NFAT signalling. Supporting a similar role for *rcan1* in fish, we found that upregulation of *rcan1* in rainbow trout coincides with hypertrophic growth and impaired cardiac performance (Johansen et al., 2017). In the current study, we did not see an increase in *rcan1* mRNA expression, indicating that up to 21 days of cortisol exposure is not sufficient to induce such pathological signalling in the rainbow trout heart. On the contrary, we observed small but not significant decreases in *rcan1* following 2 and 7 days of cortisol treatment before expression levels were slightly, but not significantly, increased after 21 days of treatment (yielding a significant interaction effect between cortisol treatment and time on *rcan1* expression).

In the time course study, we found an overall increase in relative ventricular mass (RVM) of isolated fish when combining all treatment groups, (i.e. 2, 7 and 21 days) but the increase was not significantly different between any exposure groups compared to their respective treatment controls, including following 21 days of treatment. Thus, unlike in group-reared fish where 21 days of cortisol treatment induced a 20% and significant increase in RVMs, RVMs were not increased following 21 days of treatment in isolation. Curiously, the 21 days treatment controls kept in isolation had increased RVMs compared to the 2 days treatment controls. In contrast, control fish kept in groups for 21 days, had RVMs comparable to those of 2 and 7 days treatment controls in the time-course experiment. It is difficult to explain the increase in RVMs of the 21 days treatment controls kept in isolation. It is possible that this increase is merely incidental. It could also reflect an effect of stress since social isolation has been suggested to be stressful for rainbow trout. Bernier et al. (2008) showed that plasma cortisol levels increased 24 h after transfer to social isolation (9 l compartments) but returned to control levels after 96 h. In the current study, all treatment controls (kept in 50 l aquaria), including the 21 days treatment controls, had very low cortisol levels at the times of sampling. In addition, the increment in individual feed intake during the 10 day acclimation period suggests that a potential stress response from being moved to a novel environment (from rearing tanks to individual aquaria) was reduced during acclimation. Thus, we speculate that social isolation is not necessarily stressful for a territorial species like rainbow trout. We did not, however, investigate other stress hormones or factors that could potentially be elevated by social isolation and contribute to increased RVM. For example, catecholamines (CAs) like epinephrine and nor-epinephrine as well as the monoamine serotonin (5-HT), which are also elevated by stress (Barton, 2002), have receptors in several tissues and organ systems, including the myocardium. For example, CAs are potent mediators of stress-related cardiac remodelling in mammals (Zimmer, 2003). In fish, though, the effects of CAs on the myocardium seem less pronounced (Tota et al., 2010). Like CAs, 5-HT plays a critical role in the cardiovascular system of mammals and appears to be a potent hypertrophic stimulus (Jaffr; et al., 2009; Lairez et al., 2013). To our knowledge, it is not known whether 5-HT has similar effects on the teleost heart. Whether cortisol acts in company with other signalling molecules such as CAs and 5-HT to induce cardiac remodelling in fish certainly deserves further scrutiny. After all, cortisol modulates CAs storage and release (Reid et al., 1996) and is itself modulated by 5-HT in fish (Winberg et al., 1997).

Cortisol mediates direct effects on cardiac tissue primarily via activation of the intracellular low affinity glucocorticoid (GRs) and high affinity mineralocorticoid (MR) receptors, which act as transcription factors. Although the exact mechanisms behind corticosteroid-induced remodelling are not fully elucidated, they are likely receptor-specific. For example, in mammals, corticosteroid-induced pro-hypertrophic signalling has been shown to be mediated through GR, but not MR (Ren et al., 2012) signalling, whereas corticosteroid-induced fibrotic remodelling is believed to be mediated by the MR receptor only (Funder, 2005). In the current study, the cortisol treatment had a marked effect on the expression of *mr*. More precisely, *mr* expression was downregulated following 7 days of treatment, but back to control values following 21 days of exposure. A similar pattern was observed for the mRNA expression of the two teleost *gr* receptors (*gr1* and *gr2*), although changes in expression were not statistically significant. Similar depressive effects of stress or cortisol on the expression of both *mr* and *gr*s have been described for other cortisol-sensitive tissues in rainbow trout, like the brain (Johansen et al., 2011b), liver (Vijayan et al., 2003), gills, muscle and intestine (Teles et al., 2013). The current data indicate that the myocardium is also subject to a similar autoregulation of cortisol receptors, perhaps serving to reduce tissue responsiveness to excess cortisol. The observed downregulation of *mr* was, however, not maintained throughout the treatment period perhaps indicating that such a potentially protective mechanism is temporary. Failure to maintain this protection against excess cortisol could mediate previously observed pathological effects of long-term cortisol exposure on the salmonid heart (Johansen et al., 2011a, 2017).

Since cortisol can act directly through these receptors to alter expression of target genes, it is not unreasonable to assume that such an expression pattern can also have permitted time-dependent effects of cortisol on gene expression in this study. Moreover, since the three cortisol receptors also have different affinity for or transactivational activity (*gr2* is for example activated at far lower concentrations of cortisol than *gr1*) in response to cortisol, their expression pattern can also permit dose-dependent effects of cortisol (Bury et al., 2003). We did not monitor cortisol levels throughout the experimental period and cannot elaborate on potential dose-dependent effects. From the time course experiment, however, we see that mean cortisol levels were high at all time points measured (i.e. 2, 7 and 21 days) perhaps allowing for activation of all three receptors. There was, however, large variation in plasma cortisol levels where some cortisol-treated individuals had low levels at least at the time of sampling. Thus, we cannot exclude the likely possibility that cortisol exposure varied within the cortisol-treated groups of fish.

Particularly, in the experimental setup where fish were reared in groups, feed intake could be influenced by social status. Subordinate individuals in a social hierarchy suffer from stress and elevated cortisol levels (Noakes and Leatherland, 1977; Winberg and Lepage, 1998), both of which reduces feed intake in salmonids (Barton et al., 1987; Øverli et al., 1998; Gregory and Wood, 1999). Dominant individuals can also prevent subordinate individuals from accessing feed (Øverli et al., 1998; Gilmour et al., 2005). There are, however, several factors suggesting that social stress was low in our experiment. Firstly, and perhaps most importantly, plasma cortisol levels of group-reared control fish were very low (see Fig. 1A). Secondly, all fish were closely monitored during feeding and all fish were actively seeking and consuming food following the 10 day acclimation period. Indeed, growth rates (grams of body weight gained per day) were positive for all group-reared control fish (ranging from 1.6 to 3.8 g day^−1^, data not shown) and all except one cortisol-treated fish (ranging from −3.9 to 1.3 g day^−1^, data not shown). Although there are several factors other than feed intake that influence growth rate, a positive growth rate is directly associated with food consumption.

In conclusion, we confirm that cortisol is a potent pro-hypertrophic stimulant in rainbow trout. In several aspects, cortisol-induced cardiac remodelling resembles adaptive hypertrophic growth of the rainbow trout heart (i.e. cold-induced hypertrophic growth) with marked increases in hypertrophy markers (i.e. *smlc2*, *vmhc*) and natriuretic peptides (i.e. *anp* and *bnp*) (Vornanen et al., 2005; Keen et al., 2015). It is therefore important to identify molecular mechanisms that distinguish pathological (i.e. cortisol-induced) from physiological (i.e. cold-induced) heart growth. For example, the regulation of the cardio protective natriuretic peptide, *bnp*, appears to differ between cold-induced and cortisol-induced hypertrophic growth. A failure to persistently produce and express *bnp* in the latter case could make the cortisol-exposed heart more vulnerable to the potentially deleterious consequences of elevated workload. Similarly, failure to maintain a potentially protective autoregulation of *mr* could mediate pathological effects of long-term cortisol exposure and perhaps explain time-dependent effects of cortisol on cardiac gene expression and remodelling. Importantly, our data indicate that short-term stressors and life cycle transitions associated with elevated cortisol levels can potentially impact on both hypertrophic and non-hypertrophic remodelling of the trout heart.

## MATERIALS AND METHODS

### Experimental animals

The experimental animals were juvenile rainbow trout obtained from a commercial breeder (Valdres Ørretoppdrett Røn Gård, Valdres, Norway). Experiments were conducted in March at the fish holding facilities of the Department of Biosciences at the University of Oslo. Prior to experiments, fish were held in a 1250 l holding tank (250×100×50 cm) for at least 3 weeks. The holding tank was continuously supplied with dechlorinated Oslo tap water (1000 l h^−1^) with a light regime of 12:12 h light/dark. During this period, the fish were fed once daily with commercial trout pellets (EFICO, Enviro, 920, Biomar, Brande, Denmark) corresponding to 1% of their body weight.

This work was conducted in accordance with the laws and regulations controlling experiments and procedures on live animals in Norway and was approved by the Norwegian Animal Research Authority (license number 2012/33240-4100).

### Experimental set up and procedure

For the initial cortisol exposure experiment, 32 individuals with a mean±s.d. body weight of 213.52±40.63 were taken from the holding tank, anesthetised in 0.25 mg l^−1^ MS-222 (Sigma-Aldrich, St Louis, USA) and randomly distributed to four 250 l glass aquaria (100×50×50 cm), with eight individuals per group and two duplicates per group. The aquaria were continuously aerated and supplied with dechlorinated Oslo tap water (0.25 l h^−1^, 8-9°C, 12:12 h light/dark). The fish were acclimated for 10 days. Eight days into the acclimation period, the fish were anesthetised in 0.25 mg l^−1^ MS-222 and tagged with a passive integrated transponder (PIT) tag in order to obtain data on individual fish. The fish were then transferred back to their assigned aquarium. A comparison of the body weights recorded after PIT-tagging showed that there was no significant difference in weight between the four groups (unpaired *t*-test; t=0.33, *P*=0.78). Since salmonids held together quickly establish a social hierarchy in which dominant individuals might prevent some fish from eating (Øverli et al., 1998; Gilmour et al., 2005), fish were fed at three time points each day (10:00 h, 14:00 h and 16:00 h) in order to increase the probability that all individuals had the opportunity to eat. The daily total feed given was equivalent to 0.8% of the total body weight in the aquarium, both during the acclimation and the treatment periods. All fish were actively seeking and consuming the food at the start of the experiment.

After acclimation, the aquaria were randomly assigned to either control feed (*n*=16) or cortisol-enriched feed [4 μg g^−1^ body weight (BW); *n*=16] for 21 days.

For the time course study, 32 individuals with a mean±s.d. body weight of 166 g±4.3 g were individually placed into one of four equally sized compartments in a 250 l glass aquarium (100×50×50 cm, eight aquaria total). The sides and the bottom of each aquarium were covered on the outside with black plastic film and the divisions between compartments consisted of opaque PVC-walls. The aquaria were continuously aerated and supplied with dechlorinated Oslo tap water (0.25 l min^−1^, 5-7°C, 12:12 h light/dark).

At the onset of the experiment, fish were taken from the holding tank and mildly anesthetised in a bath of 0.25 g l^−1^ MS-222. They were subsequently weighed, moved to the individual compartments and allowed to acclimate for 10 days prior to treatment. Individual housing of fish in this experiment allowed for a more thorough observation of food intake to make sure that all experimental fish received the cortisol treatment. During acclimation, the fish were fed commercial trout pellets once daily between 10:00 h and 14:00 h. The fish were offered food pellets equivalent to 0.8% of their body weight and individual food intake was registered daily. Any uneaten food was removed directly after feeding. Mean±s.e.m. daily feed intake increased steadily throughout the acclimation period and went from 21.87±3.95% of daily ration on the first day of acclimation to 63.3±3.94% of daily ration on day 10 (*n*=48, pooled data from all treatment groups). During the treatment period, fish were fed with a daily ration of either control feed or cortisol-enriched (4 μg g^−1^ BW) feed for 2, 7 or 21 days. Individually housed fish from the same aquarium were given the same diet. Accordingly, the aquaria were assigned at random to the following diets; control 2 days (*n*=8), cortisol 2 days (*n*=8), control 7 days (*n*=8), cortisol 7 days (*n*=8), control 21 days (*n*=8) and cortisol 21 days (*n*=8). Initial body weights did not differ significantly between the groups [ANOVA; *F*_(5,42)_=0.55, *P*=0.74]. Three fish that did not eat during acclimation were excluded from the experiment.

### Sampling

In both experiments, sampling was conducted between 09:00 and 13:00 h the day after the last feeding. Fish were taken from their aquarium in random order and anesthetised in a bath containing a lethal dose of 1 mg l^−1^ MS-222. The fish were weighed and a blood sample was collected from the caudal vein before they were decapitated. The blood samples were centrifuged for 5 min at 4°C, 8000 ***g***. Plasma was frozen and stored at −20°C for later analysis of cortisol levels. Hearts were surgically excised and the bulbus and atrium removed. Ventricles were blotted dry for blood, weighed on a precision weight and RVM [RVM=(ventricle wet mass *M*_b_^−1^)*100], was calculated. The ventricles were then cut into two approximately equal halves, placed in 1.5 ml RNAlater^®^ solution (Ambion, Austin, USA) and left at room temperature for 24 h (according to the manufacturer's recommendations) before stored at −20°C.

### Preparation of experimental feed

The experimental diet was prepared by dissolving cortisol (hydrocortisone powder, Sigma-Aldrich) in rapeseed oil by the use of a magnetic stirrer. Specifically, 15 mg of oil containing 500 mg cortisol was then applied to 1 kg prefabricated pellets inside a vacuum coater. The container had two valves; an intake coupled to a vacuum pump and an outlet which let the air out. In order to draw the cortisol into the pellets, a negative pressure of 0.9 bar was applied, at which point the valve connected to the vacuum pump was closed and feed was mixed by shaking the container by hand ten times. Thereafter, the valve was opened to let in some air and closed again before the container was shaken ten more times. This was repeated once more. Thereafter the whole procedure (applying vacuum, letting in air and shaking) was repeated twice. Control feed was prepared in the same way but with rapeseed oil only.

### RNA extraction and quantitative real time-PCR analysis

Total RNA was extracted from 12 randomly selected ventricles per group from group-reared fish and all ventricles from the time-course experiment using Trizol reagent (Invitrogen) according to the manufacturer's protocol. Extractions were performed in random order. Briefly, the tissue samples were homogenised in Trizol reagent at a ratio of 15 μl Trizol mg^−1^ tissue. The RNA was treated with DNase using the TURBO DNA-free Kit (Invitrogen). Purified RNA was quantified using the NanoDrop ND-2000 UV-Vis Spectrophotometer (Thermo Fisher Scientific). RNA quality was assessed using the 2100 Bioanalyzer (Agilent Technologies Inc., Santa Clara, USA). RNA integrity numbers (RINs) for the tissue samples ranged from 8.40 to 10, with an average of 9.3±0.03 (mean±s.e.m.), confirming excellent RNA quality. A total of 2 μg RNA was reverse transcribed into cDNA using oligo(dT)18-20 primers (Invitrogen) and the SuperScript III Reverse Transcriptase Kit (Invitrogen).

Quantitative real-time PCR (qRT-PCR) reactions were performed with the LightCycler480 Real-Time PCR System (Roche Diagnostics, Penzberg, Germany), using the LightCycler 480 SYBR Green 1 Master mix (Roche Diagnostics) with 3 μl 1:25× diluted cDNA, 1 μM of each primer, for a total reaction volume of 10 μl. All reactions were run in duplicates on different plates. The crossing point (Cp) values were calculated by the LightCycler480 software. The efficiency of each reaction was calculated with the LinReg software (version 2012.1). Relative mRNA abundance was calculated from the following formula: (Ref_ECp_/GOI_ECp_), where E is the mean efficiency for the primer pair, Cp is the mean Cp value for the two duplicate qPCR reactions, Ref is the reference gene and GOI is the gene of interest.

Gene specific primer sequences for rainbow trout *β-actin*, slow myosin light chain 2 (*smlc2*), ventricular myosin heavy chain (*vmhc*), regulator of calcineurin 1 (*rcan1*), atrial natriuretic peptide (*anp*), B-type natriuretic peptide (*bnp*), proliferating cell nuclear antigen (*pcna*), vascular endothelial growth factor (*vegf*), mineralocorticoid receptor (*mr*), glucocorticoid receptor 1 (*gr1*) and glucocorticoid receptor 2 (*gr2*) were designed and published previously (Johansen et al., 2011a, 2017). Primers for alpha-skeletal actin 1 (*acta1*) were designed using the web-based Primer3 program (http://frodo.wi.mit.edu/primer3/) and synthesised by Invitrogen. The *acta1* (GenBank accession number: AF503211.2) sequence was retrieved from the NCBI database (http://www.ncbi.nlm.nih.gov/). A minimum of five primer pairs were designed at exon junctions and the primers showing the lowest crossing point values, a single peak melting curve and amplification of the right amplicon were chosen (forward primer: 5′-CCTCATAGATGGGGACGTTG-3′, reverse primer: 5′-CCAAGGCCAACAGAGAGAAG-3′). The qPCR product was sequenced to verify that the primers amplified the right cDNA. The housekeeping gene *β-actin* was used as reference gene as this has previously been evaluated to be a suitable control gene in studies on cortisol-induced cardiac remodelling (Johansen et al., 2011a, 2017).

### Radioimmunoassay quantification of plasma cortisol

Cortisol was measured in plasma from a selection of group-reared individuals (eight randomly chosen individuals from each group) and from all experimental fish kept in isolation. Plasma cortisol was analysed using a radioimmunoassay as described by Johansen et al. (2017). The lower detection limit of the assay was 0.19 ng ml^−1^. For individuals where the plasma cortisol levels were below this limit, the level was set to 0.2 ng ml^−1^. The upper limit was 655 ng ml^−1^. For individuals that displayed higher plasma cortisol levels than this, the level was set to 650 ng ml^−1^.

### Statistical analysis

Values are presented as mean±s.e.m. All statistical analyses were performed using GraphPad Prism7 (GraphPad Software). Data from fish kept in groups were analysed by unpaired *t*-tests with Welch's correction for unequal variance when relevant (*n*=12). Data from the time course experiment were analysed by two-way ANOVA to examine whether cortisol treatment, treatment period or the interaction between these two independent variables had an effect on plasma cortisol levels, with RVM and the expression of remodelling markers as dependent variables. The two-way ANOVA was then followed by Sidak's planned comparison test (with adjusted alpha) to be able to assess differences between treatment groups within each time point. In the time course experiment, data on plasma cortisol levels, *smlc2*, *mr*, *anp*, *bnp* and *pcna* did not show variance homogeneity (Bartlett's test) and were log-transformed prior to analysis. Differences were considered significant for *P*<0.05.

## References

[BIO037853C1] Barron M. G. (1986). Endocrine control of smoltification in anadromous salmonids. *J. Endocrinol.* 108, 313-319. 10.1677/joe.0.1080313 3005463

[BIO037853C2] Barton B. A. (2002). Stress in fishes: a diversity of responses with particular reference to changes in circulating corticosteroids. *Integr. Comp. Biol.* 42, 517-525. 10.1093/icb/42.3.517 21708747

[BIO037853C3] Barton B. A. and Peter R. E. (1982). Plasma cortisol stress response in fingerling rainbow trout, *Salmo gairdneri Richardson*, to various transport conditions, anaesthesia, and cold shock. *J. Fish. Biol.* 20, 39-51. 10.1111/j.1095-8649.1982.tb03893.x

[BIO037853C4] Barton B. A., Schreck C. B., Ewing R. D., Hemmingsen A. R. and Patiño R. (1985). Changes in plasma cortisol during stress and smoltification in coho salmon, *Oncorhynchus kisutch*. *Gen. Comp. Endocrinol.* 59, 468-471. 10.1016/0016-6480(85)90406-X 2995200

[BIO037853C5] Barton B. A., Schreck C. B. and Barton L. D. (1987). Effects of chronic cortisol administration and daily acute stress on growth, physiological conditions, and stress responses in juvenile rainbow trout. *Dis. Aquat. Organ.* 2, 173-185. 10.3354/dao002173

[BIO037853C6] Bernier N. J., Alderman S. L. and Bristow E. N. (2008). Heads or tails? Stressor-specific expression of corticotropin-releasing factor and urotensin I in the preoptic area and caudal neurosecretory system of rainbow trout. *J. Endocrinol.* 196, 637-648. 10.1677/JOE-07-0568 18310459

[BIO037853C7] Bradley R., Markson L. M. and Bailey J. (1981). Sudden death and myocardial necrosis in cattle. *J. Pathol.* 135, 19-38. 10.1002/path.1711350104 7299529

[BIO037853C8] Bury N. R., Sturm A., Le Rouzic P., Lethimonier C., Ducouret B., Guiguen Y., Robinson-Rechavi M., Laudet V., Rafestin-Oblin M. E. and Prunet P. (2003). Evidence for two distinct functional glucocorticoid receptors in teleost fish. *J. Mol. Endocrinol.* 31, 141-156. 10.1677/jme.0.0310141 12914532

[BIO037853C9] Carruth L. L., Dores R. M., Maldonado T. A., Norris D. O., Ruth T. and Jones R. E. (2000). Elevation of plasma cortisol during the spawning migration of landlocked kokanee salmon (*Oncorhynchus nerka kennerlyi*). *Comp. Biochem. Physiol. C.* 127, 123-131. 10.1016/S0305-0491(00)00245-5 11083023

[BIO037853C10] Clark R. J. and Rodnick K. J. (1998). Morphometric and biochemical characteristics of ventricular hypertrophy in male rainbow trout (*Oncorhynchus mykiss*). *J. Exp. Biol.* 201, 1541-1552. 9556537 10.1242/jeb.201.10.1541

[BIO037853C11] Farrell A. P., Hammons A. M., Graham M. S. and Tibbits G. F. (1988). Cardiac growth in rainbow trout, *Salmo gairdneri*. *Cana. J. Zool.* 66, 2368-2373. 10.1139/z88-351

[BIO037853C12] Franklin C. E. and Davie P. S. (1992). Sexual maturity can double heart mass and cardiac power output in male rainbow trout. *J. Exp. Biol.* 171, 139-148.

[BIO037853C13] Frohlich E. D. (1990). Left ventricular hypertrophy: an independent factor of risk. *Cardiovasc. Clin.* 20, 85-94. 2140072

[BIO037853C14] Funder J. W. (2005). RALES, EPHESUS and redox. *J. Steroid. Biochem. Mol. Biol.* 93, 121-125. 10.1016/j.jsbmb.2004.12.010 15860254

[BIO037853C15] Gamperl A. K. and Farrell A. P. (2004). Cardiac plasticity in fishes: environmental influences and intraspecific differences. *J. Exp. Biol.* 207, 2539-2350 10.1242/jeb.01057 15201287

[BIO037853C16] Gilmour K. M., Dibattista J. D. and Thomas J. B. (2005). Physiological causes and consequences of social status in salmonid fish. *Integr. Comp. Biol.* 45, 263-273. 10.1093/icb/45.2.263 21676770

[BIO037853C17] Graham M. S. and Farrell A. P. (1989). The effect of temperature acclimation and adrenaline on the performance of a perfused trout heart. *Physiol. Zool.* 62, 38-61. 10.1086/physzool.62.1.30159997

[BIO037853C18] Gregory T. R. and Wood C. M. (1999). The effects of chronic plasma cortisol elevation on the feeding behaviour, growth, competitive ability, and swimming performance of juvenile rainbow trout. *Physiol. Biochem. Zool.* 72, 286-295. 10.1086/316673 10222323

[BIO037853C19] Hama N., Itoh H., Shirakami G., Nakagawa O., Suga S.-I., Ogawa Y., Masuda I., Nakanishi K., Yoshimasa T., Hashimoto Y. et al. (1995). Rapid ventricular induction of brain natriuretic peptide gene expression in experimental acute myocardial infarction. *Circulation* 92, 1558-1564. 10.1161/01.CIR.92.6.1558 7664440

[BIO037853C20] Jaffré F., Bonnin P., Callebert J., Debbabi H., Setola V., Doly S., Monassier L., Mettauer B., Blaxall B. C., Launay J.-M. et al. (2009). Serotonin and angiotensin receptors in cardiac fibroblasts coregulate adrenergic-dependent cardiac hypertrophy. *Circ. Res.* 104, 113-123. 10.1161/CIRCRESAHA.108.180976 19023134

[BIO037853C21] Johansen I. B., Lunde I. G., Røsjø H., Christensen G., Nilsson G. E., Bakken M. and Øverli Ø. (2011a). Cortisol response to stress is associated with myocardial remodeling in salmonid fishes. *J. Exp. Biol.* 214, 1313-1321. 10.1242/jeb.053058 21430209

[BIO037853C22] Johansen I. B., Sandvik G. K., Nilsson G. E., Bakken M. and Øverli Ø. (2011b). Cortisol receptor expression differs in the brains of rainbow trout selected for divergent cortisol responses. *Comp. Biochem. Physiol. D.* 6, 126-132. 10.1016/j.cbd.2010.11.002 21220219

[BIO037853C23] Johansen I. B., Sandblom E., Skov P. V., Gräns A., Ekström A., Lunde I. G., Vindas M. A., Zhang L., Höglund E., Frisk M. et al. (2017). Bigger is not better: cortisol-induced cardiac growth and dysfunction in salmonids. *J. Exp. Biol.* 220, 2545-2553. 10.1242/jeb.135046 28476893

[BIO037853C24] Keen A. N., Fenna A. J., McConnell J. C., Sherratt M. J., Gardner P. and Shiels H. A. (2015). The dynamic nature of hypertrophic and fibrotic remodeling of the fish ventricle. *Front. Physiol.* 6, 427. 26834645 10.3389/fphys.2015.00427 PMC4720793

[BIO037853C25] Lairez O., Cognet T., Schaak S., Calise D., Guilbeau-Frugier C., Parini A. and Mialet-Perez J. (2013). Role of serotonin 5-HT2A receptors in the development of cardiac hypertrophy in response to aortic constriction in mice. *J. Neural. Transm. (Vienna).* 120, 927-935. 10.1007/s00702-013-1011-3 23543114

[BIO037853C26] Lear W. and Boer P. H. (1995). Rapid activation of the type B versus type A natriuretic factor gene by aortocaval shunt induced cardiac volume overload. *Cardiovasc. Res.* 29, 676-681. 10.1016/S0008-6363(96)88640-8 7606757

[BIO037853C27] Lloyd-Jones D. M., Larson M. G., Leip E. P., Beiser A., D'Agostino R. B., Kannel W. B., Murabito J. M., Vasan R. S., Benjamin E. J. and Levy D. (2002). Lifetime risk for developing congestive heart failure: the Framingham Heart Study. *Circulation.* 106, 3068-3072. 10.1161/01.CIR.0000039105.49749.6F 12473553

[BIO037853C28] Maule A. G., Schreck C. B., Bradford C. S. and Barton B. A. (1988). Physiological effects of collecting and transporting emigrating juvenile Chinook salmon past dams on the Columbia river. *Trans. Am. Fisher. Soc.* 117, 245-261. 10.1577/1548-8659(1988)117<0245:PEOCAT>2.3.CO;2

[BIO037853C29] Maxwell M. H. and Robertson G. W. (1998). UK survey of broiler ascites and sudden death syndromes in 1993. *Br. Poult. Sci.* 39, 203-215. 10.1080/00071669889132 9649872

[BIO037853C30] Messerli F. H. and Ketelhut R. (1991). Left ventricular hypertrophy: an independent risk factor. *J. Cardiovasc. Pharmacol.* 17, S59-S66. 10.1097/00005344-199117040-00014 1726010

[BIO037853C31] Noakes D. L. G. and Leatherland J. F. (1977). Social dominance and interrenal cell activity in rainbow trout, *Salmo gairdneri* (Pisces, Salmonidae). *Environ. Biol. Fish.* 2, 131-136. 10.1007/BF00005368

[BIO037853C32] Poppe T. T., Johansen R., Gunnes G. and Tørud B. (2003). Heart morphology in wild and farmed Atlantic salmon *Salmo salar* and rainbow trout *Oncorhynchus mykiss*. *Dis. Aquat. Organ.* 57, 103-108. 10.3354/dao057103 14735927

[BIO037853C33] Poppe T. T., Johansen R. and Torud B. (2002). Cardiac abnormality with associated hernia in farmed rainbow trout *Oncorhynchus mykiss*. *Dis. Aquat. Organ.* 50, 153-155. 10.3354/dao050153 12180706

[BIO037853C34] Poppe T. T., Taksdal T. and Bergtun P. H. (2007). Suspected myocardial necrosis in farmed Atlantic salmon, *Salmo salar L*.: a field case. *J. Fish. Dis.* 30, 615-620. 10.1111/j.1365-2761.2007.00841.x 17850577

[BIO037853C35] Reid S. G., Vijayan M. M. and Perry S. F. (1996). Modulation of catecholamine storage and release by the pituitary-interrenal axis in the rainbow trout, *Oncorhynchus mykiss*. *J. Comp. Physiol. B.* 165, 665-676. 10.1007/BF00301135 8882512

[BIO037853C36] Ren R., Oakley R. H., Cruz-Topete D. and Cidlowski J. A. (2012). Dual role for glucocorticoids in cardiomyocyte hypertrophy and apoptosis. *Endocrinology* 153, 5346-5360. 10.1210/en.2012-1563 22989630 PMC3473206

[BIO037853C37] Schmidt P. J. and Idler D. R. (1962). Steroid hormones in the plasma of salmon at various states of maturation. *Gen. Comp. Endocrinol.* 2, 204-214. 10.1016/0016-6480(62)90005-9 13908787

[BIO037853C38] Shih Y.-H., Zhang Y., Ding Y., Ross C. A., LI H., Olson T. M. and Xu X. (2015). Cardiac transcriptome and dilated cardiomyopathy genes in zebrafish. *Circ. Cardiovasc. Genet.* 8, 261-269. 10.1161/CIRCGENETICS.114.000702 25583992 PMC4406804

[BIO037853C39] Shimizu I. and Minamino T. (2016). Physiological and pathological cardiac hypertrophy. *J. Mol. Cell. Cardiol.* 97, 245-262. 10.1016/j.yjmcc.2016.06.001 27262674

[BIO037853C40] Strange R. J., Schreck C. B. and Ewing R. D. (1978). Cortisol concentrations in confined juvenile Chinook salmon (*Oncorhynchus tshawytscha*). *Trans. Am. Fisher. Soc.* 107, 812-819. 10.1577/1548-8659(1978)107<812:CCICJC>2.0.CO;2

[BIO037853C41] Teles M., Tridico R., Callol A., Fierro-Castro C. and Tort L. (2013). Differential expression of the corticosteroid receptors GR1, GR2 and MR in rainbow trout organs with slow release cortisol implants. *Comp. Biochem. Physiol. A.* 164, 506-511. 10.1016/j.cbpa.2012.12.018 23277222

[BIO037853C42] Tota B., Cerra M. C. and Gattuso A. (2010). Catecholamines, cardiac natriuretic peptides and chromogranin A: evolution and physiopathology of a ‘whip-brake’ system of the endocrine heart. *J. Exp. Biol.* 213, 3081-3103. 10.1242/jeb.027391 20802109

[BIO037853C43] Vijayan M. M., Raptis S. and Sathiyaa R. (2003). Cortisol treatment affects glucocorticoid receptor and glucocorticoid-responsive genes in the liver of rainbow trout. *Gen. Comp. Endocrinol.* 132, 256-263. 10.1016/S0016-6480(03)00092-3 12812773

[BIO037853C44] Vornanen M., Hassinen M., Kokskinen H. and Krasnov A. (2005). Steady-state effects of temperature acclimation on the transcriptome of the rainbow trout heart. *Am. J. Physiol. Regul. Integr. Comp. Physiol.* 289, R1177-R1184. 10.1152/ajpregu.00157.2005 15932967

[BIO037853C45] Weeks K. L. and McMullen J. R. (2011). The athlete's heart vs. the failing heart: can signaling explain the two distinct outcomes? *Physiology* 26, 97-105. 10.1152/physiol.00043.2010 21487028

[BIO037853C46] Wilkins B. J., Dai Y.-S., Bueno O. F., Parsons S. A., Xu J., Plank D. M., Jones F., Kimball T. R. and Molkentin J. D. (2004). Calcineurin/NFAT coupling participates in pathological, but not physiological, cardiac hypertrophy. *Circ. Res.* 94, 110-118. 10.1161/01.RES.0000109415.17511.18 14656927

[BIO037853C47] Winberg S. and Lepage O. (1998). Elevation of brain 5-HT activity, POMC expression, and plasma cortisol in socially subordinate rainbow trout. *Am. J. Physiol.* 274, R645-R654. 10.1152/ajpcell.1998.274.3.C645 9530229

[BIO037853C48] Winberg S., Nilsson A., Hylland P., Söderström V. and Nilsson G. E. (1997). Serotonin as a regulator of hypothalamic-pituitary-interrenal activity in teleost fish. *Neurosci. Lett.* 230, 113-116. 10.1016/S0304-3940(97)00488-6 9259477

[BIO037853C49] Wolf K., Kurtz A., Pfeifer M., Höcherl K., Riegger G. A. and Krämer B. K. (2001). Different regulation of left ventricular ANP, BNP and adrenomedullin mRNA in the two-kidney, one-clip model of renovascular hypertension. *Pflügers. Archiv.* 442, 212-217. 10.1007/s004240100533 11417216

[BIO037853C50] Zimmer H. G. (2003). Catecholamines and cardiac remodeling. In *Cardiac Remodeling and Failure* (ed. Singal P. K., Dixon I. M. C., Kirschenbaum L. A. and Dhalla N. S.), pp. 293-304. Boston, MA: Springer US.

[BIO037853C51] Øverli Ø., Winberg S., Damsgård B. and Jobling M. (1998). Food intake and spontaneous swimming activity in Arctic char (*Salvelinus alpinus*): role of brain serotonergic activity and social interactions. *Can. J. Zool.* 76, 1366-1370. 10.1139/z98-050

